# Intensive Care in a Patient with Toxic Epidermal Necrolysis

**DOI:** 10.1155/2017/3246196

**Published:** 2017-11-01

**Authors:** J. Wallenborn, M. Fischer

**Affiliations:** ^1^Department of Anesthesiology and Intensive Care Medicine, HELIOS Klinikum Aue, Gartenstraße 6, 08280 Aue, Germany; ^2^Department of Anesthesiology and Intensive Care Medicine, Universitätsklinikum Carl Gustav Carus, Technische Universität Dresden, Fetscherstraße 74, 01307 Dresden, Germany; ^3^Department of Dermatology and Venerology, HELIOS Klinikum Aue, Gartenstraße 6, 08280 Aue, Germany; ^4^Department of Dermatology and Venerology, Martin-Luther-Universität Halle-Wittenberg, Ernst-Grube-Straße 40, 06097 Halle, Germany

## Abstract

Toxic epidermal necrolysis (TEN) is a serious adverse drug reaction with high lethality, which usually requires intensive-medical care. A 44-year-old man developed generalized exanthema with increasing exfoliation and mucosal involvement after taking allopurinol, ibuprofen, and etoricoxib. The clinical diagnosis of TEN was histologically confirmed. Prednisolone therapy with 3 mg/kg body weight (BW) was not able to prevent further progress to finally 80% of the body surface, and infliximab 5 mg/kg BW was given as a single dose. This prevented further progression of the TEN. Despite marked improvement in skin findings, the ICU stay was prolonged by a complex analgosedation, transient kidney failure, volume management, positioning therapy, and vegetatively impeded weaning. Moreover, there was colonization with multiresistant bacteria (MRSA and VRE). Nonetheless, the patient could be restored to health and was released after four weeks. Infliximab seems to be effective in the treatment of TEN, especially in cases of rapid progression. Moreover, patients with TEN are difficult to handle in intensive-medical care, whereby attention should especially be paid to sufficient pain therapy, and the positioning of the patient is a particular challenge.

## 1. Introduction

In Europe, between 2.5% and 10.6% of all in-hospital treatment cases are ascribable to adverse drug reactions [1, 2], whereby the skin is affected in only 2–5‰ of the cases. Drug-related exanthemas which require intensive-medical care or which are life-threatening are even less frequent. While nearly all affected patients survive maculopapulous or maculourticarial exanthemas, lethality of up to 10% is reported for hypersensitivity reactions like Drug Rash with Eosinophilia and Systemic Symptoms (DRESS) [3]. Depending on the spread, toxic epidermal necrolysis (TEN) has by far the highest lethality [4]. The intensive-medical care of these patients is very demanding and characterized by various particular features.

## 2. Case Presentation

**Figure 1:**
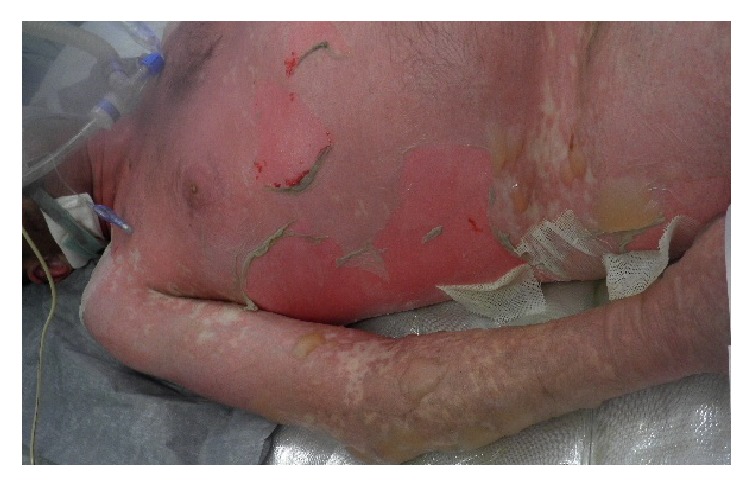
Large-area exfoliation in toxic epidermal necrolysis.

**Figure 2:**
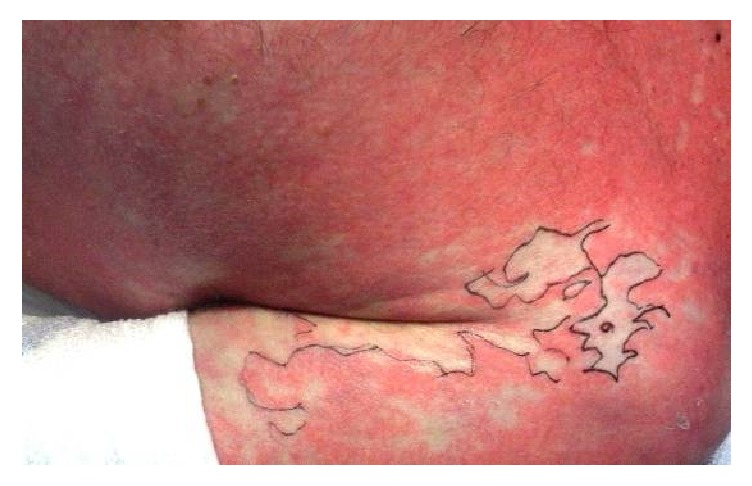
Toxic epidermal necrolysis. Cessation of progression after infliximab.

Long-term therapy with allopurinol and etoricoxib, with ibuprofen as needed, was initiated due to arthralgia in hyperuricemia in a 44-year-old man, who had been otherwise healthy up to then. Three weeks later, an itchy patch of erythema developed on his back, which spread exanthematically over the entire trunk and extremities during the following 48 hours. Withdrawal of the three cited medications and out-patient administration of prednisolone (1 mg/kg BW/die) could not prevent further progression. On admission to hospital, symmetrically distributed, confluent livid-erythematous exanthema was seen on the trunk and face, with multiple flaccid blisters of amber-colored content. Initially, ca. 50% of the body surface (BOS) was affected. The Nikolski I-Phenomenon was positive. The oral mucosa showed multiple erosions with whitish coating buccal and in the vestibulum oris. The entire prolabium was erosive and covered with scabs. Moreover, there was pronounced conjunctivitis. The SCORTEN [5] on admission was 2 points (Table 1); the risk of mortality was thus 12%. At the time of admission, the CRP was 53.4 mg/l (Norm: <5 mg/l); Leukocytes and procalcitonin were in the normal range. Due to his increasingly worse general condition, the patient was transferred to Intensive Care where initially treatment with prednisolone 3 mg/kg BW was initiated. However, within the following 48 hours, there was additional spread of the exanthema, and finally ca. 80% of the body surface was affected (Figure 1). For this reason, prednisolone was withdrawn and infliximab administered in a single dose of 5 mg/kg BW. This resulted within 6 hours in a complete stop of the exanthema progression (Figure 2). However, during the following days, partial exfoliation was seen within the erythema visible at the start of therapy. Local therapy consisted of application of gels containing polihexanide and bland gauze bandages. Moreover, the patient was bedded on a glass bead bed (Pearls® AFT, Hill-Rom GmbH, Witten, Germany) on Metalline®-Foil. The Air Fluidised Therapy with this special bed supports healing of such pronounced skin lesions by means of microclimatization, draining of secretions, and marked reduction of shear and friction, as well as pressure on the skin from the covers. Bandages were changed daily by the same team maintaining reverse isolation.

Due to a positive blood culture with proof of* Staphylococcus lugdunensis* and* Staphylococcus epidermidis* with concurrent elevated temperature (39.1°C), systemic antibiotic therapy with tazobactam and clindamycin was started on the 11th day of treatment and continued for 5 days. The PCT remained in the normal range (maximum value: 0.28 *µ*g/l (Norm: <0.5 *µ*g/l)) during the entire hospitalization period. In the regularly performed microbiological skin swabs, methicillin-resistant* Staphylococcus aureus* (MRSA) and vancomycin-resistant Enterococci (VRE) in swabs from the angle of the mouth were found starting on the 15th day of treatment.

Due to severe pain (NAS = 8–10) analgosedation was given during the entire intensive-medical treatment phase, beginning initially with piritramide (Dipidolor®, 5 mg-Boli) and lorazepam (Tavor®, 0.5 mg-Boli). The further course was characterized by prerenal kidney failure and respiratory exhaustion. Maintaining the equilibrium of the fluid household was impeded by the losses via the large skin defects, which were impossible to measure. Efforts were oriented to extensive hemodynamic monitoring (PiCCO advanced) and repetitive albumin measurements. Intubation with controlled respiration in the BiPAP mode was required starting on the 4th day due to involvement of the oral mucosa with increasing difficulty swallowing. The analgosedation was therefore switched to continuous application of sufentanil (Sufenta®: 250 *µ*g/50 ml at 2–8 ml/h) and propofol 2% (Disoprivan®: 1000 mg/50 ml at 4–10 ml/h). After only 48 hours, propofol was replaced by midazolam (Dormicum®: 90 mg/50 ml at 2–8 ml/h) and additional ketamine (Ketanest: 250 mg/50 ml at 4–8 ml/h) to achieve adequate analgesia. Repeated states of restlessness and hypertensive dysregulation up to 210 mmHg both under analgosedation and in the daily withdrawal attempts were terminated with urapidil or ß-blockers and the analgosedation expanded by additional administration of dexmedetomidine (Dexdor®: 1000 *µ*g/50 ml at 1.5–4 ml/h). This unstable state lasted for a total of ten days. Only then could weaning from the respirator be successfully initiated and the analgosedation tapered off. Extubation of the responsive patient under reduced continuing analgosedation was successfully done on the 18th day of treatment. The further course was characterized by intensive physiotherapy to relieve the prominent muscle weakness following long-term sedation and critical-illness polyneuropathy (CIP). Immediately after extubation, the patient could only make weak arm movements; in the course of the first week, the patient could be mobilized to standing and walking.

**Figure 3:**
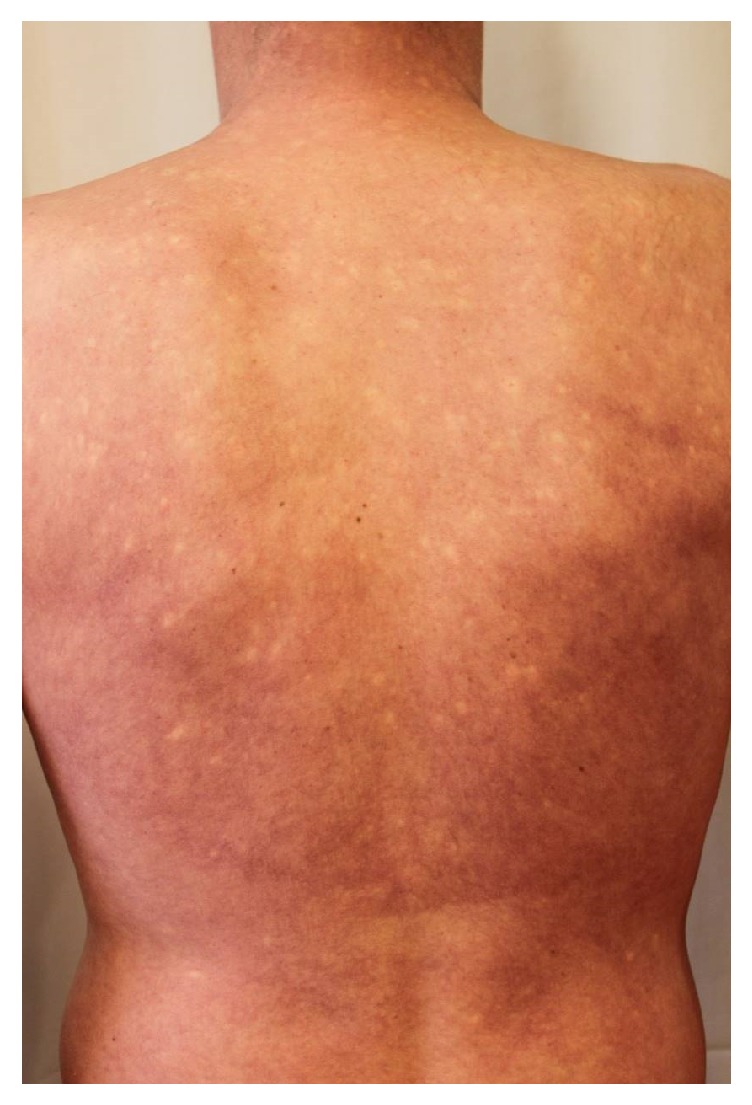
Findings on discharge. Complete healing under postinflammatory hyperpigmentations.

Under this complex treatment, there was full reepithelization of the skin with postinflammatory hyperpigmentations within four weeks (Figure 3), successful weaning, and mobilization. After reepithelization was complete, neither VRE nor MRSA were found on the skin.

## 3. Discussion

With a mortality between 39% to 48%, TEN is among the few acute life-threatening skin diseases [4, 6]. Though many drugs can induce TEN, the most common elicitors are antibiotics, anticonvulsives, nonsteroidal antirheumatics, and allopurinol [4, 6]. The diagnosis is made clinical predominantly. Nonetheless some other dermatoses with epithelial defects (staphylococcal scalded skin syndrome, pemphigus vulgaris, pemphigus foliaceus, acute generalized exanthematic pustulosis, and acute Graft-versus-Host-disease) should be taken into account. Therefore, a histological proof is mandatory.

Various mediators are involved in the apoptotic process of TEN. Granulysin is known to play a major role in inducing necrosis of keratinocytes both in* in vitro* and in a mouse model [6]. Other cytokines, especially tumor necrosis factor-alpha (TNF-*α*), are also important in the underlying cytotoxicity reaction. For example, TNF-*α* in high concentrations has been demonstrated in the blister fluid of patients with TEN [7]. Recent studies were also able to show that TNF-*α* in combination with *γ*-Interferon effects stimulation of inducible NO-synthase. The resultant NO is cytotoxic for keratinocytes [8]. The good response to the administration of corresponding biologics also illustrates a role of TNF-*α* in TEN. Since it was first described [9], prompt efficacy of both infliximab and etanercept has been repeatedly described in nearly twenty case reports and smaller case studies [10, 11]. However, no survival advantage for the administration of infliximab in combination with N-acetylcysteine over N-acetylcysteine alone could be demonstrated in a Proof-of-Concept study on ten TEN patients [12]. Here, in addition to the small number of cases, it must also be remembered that the patients in the Proof-of-Concept study were not treated until a relatively late stage of the disease. Medication administration began on average about seven days after onset of symptoms. At that point, the TNF-*α*-dependent apoptosis/necrosis has possibly already occurred and can no longer be decisively influenced by the administration of infliximab. This assumption is supported by the clinical experience that although administration of infliximab leads to prompt stopping of further area expansion of TEN, erythematous areas which are already visible may still show exfoliation. Apparently the already-initiated apoptosis cannot be reversed. This points to a particular importance of TNF-*α* in early and active phases of TEN and thus for early therapy with TNF-*α*-blockers in TEN.

The stopping of the area expansion observed under infliximab can, independent of overall survival, contribute, in any case, to the reduction of complications and facilitate in-hospital care. The expected cost reductions from new biosimilars to infliximab would also reduce the economic burden of therapy.

The intensive-medical care of TEN patients is particularly complex. Thus, in the present case, the need for analgosedation greatly exceeded the commonly applied substances and dosages [13]. In addition to administration of propofol and sufentanil, the application of ketamine and dexmedetomidine was necessary. This condition still remained during the phase of beginning reepithelization and prolonged the stay in the ICU. The cause of the high need for therapy is unclear, but it does underline the high expense of care for patients with TEN.

Due to the large wound area, there is a high sepsis risk in patients with TEN. The frequency of sepsis is estimated at 20% [14]. Kidney failure is described in 10% of the cases and respiratory failure and pneumonia in 48% of the patients. Interestingly, the bacteria found in the skin swabs of TEN patients with infectious complications have no or only slight predictive value [15], although, as in our case, there is increased skin colonization with multiresistant bacteria [11]. An initial antibiotic prophylaxis therefore does not appear justified. In response to the skin bacteria* Staphylococcus lugdunensis* and* Staphylococcus epidermidis* found in the blood culture, 5-day intravenous antibiotic therapy was administered based on the increased fever and leukocytosis. On the other hand, systemic antibiotic therapy was not given in response to the proven MRSA, but only local antiseptic cleansing and bandages. This makes an intensive-medical difference from burn patients clear. Where damage to deeper skin layers is the starting point for septic complications in burn patients [16], the immunologically caused destruction of the epidermis apparently does not lead to a clinically relevant infection focus for sepsis. This observation has repeatedly elicited discussion of whether treating of patients with large-area exfoliation in normal wards is justified with appropriately intensified care [17]. The answer can only be found as a single-case decision depending on the extent of the wound area. However, the challenges for intensive-medical care outlined here indicate that treatment of TEN patients usually requires intensive therapy (Table 2).

Positioning was in a special bed because of the desired minimal mechanical stress to the skin. This makes positioning on an air cushion with concurrent drainage of secretions and temperature regulation possible. However, the nearly pressure-free positioning leads to the negative effect of loss of depth sensitivity, and, in the case cited, muscular weakness after days of immobilization and onset of CIP were correspondingly pronounced. Intensive physiotherapy supported rapid reconvalescence.

Recent studies [18] pointed out that cyclosporine reduces the mortality of TEN compared with supportive care. Nonetheless TNF-*α*-blockers like infliximab seem to be effective especially in cases with rapid progression.

## Figures and Tables

**Table 1:** Prognosis score for TEN (SCORTEN) [5]. Determination on admission. A point is given for a fulfilled criterion. Rating of mortality: ≤1 points 3%, 2 points 12%, 3 points 35%, 4 points 58%, and ≥5 points 90%.

Parameter SCORTEN	Points
Age ≥ 40	1
Pulse ≥ 120/min	0
Malignoma	0
Affected body surface ≥ 10%	1
Urea > 10 mmol/l	0
Bicarbonate (HCO_3_) < 20 mmol/l	0
Glucose > 14 mmol/l	0

**Table 2:** Supportive care in TEN.

Local therapy with polihexanide gel and gauze bandages
Reverse isolation
No antibiotic prophylaxis
Sequential microbiological screening, targeted antibiotic therapy for sepsis
Analgosedation
Volume therapy, albumin substitution
In complications: intubation and respiration, renal substitution therapy, sepsis therapy
Positioning therapy
Physiotherapy, ergotherapy, logopedics
